# Optimism about Measuring Animal Feelings

**DOI:** 10.1007/s41649-023-00244-7

**Published:** 2023-03-15

**Authors:** Heather Browning, Walter Veit

**Affiliations:** 1 University of Southampton, Southampton, UK; 2 University of Bristol, Bristol, UK

**Keywords:** Animal sentience, Animal pain, Animal welfare, Animal ethics, Consciousness studies

## Abstract

While animal sentience research has flourished in the last decade, scepticism about our ability to accurately measure animal feelings has unfortunately remained fairly common. Here, we argue that evolutionary considerations about the functions of feelings will give us more reason for optimism and outline a method for how this might be achieved.

Animal sentience research has come to grown into something like a new discipline within the last decade. Yet, scepticism about our capacity to measure animal feelings has remained a widespread position. In a recent review of the field, Rowan et al. (2021) have provided a thorough analysis of the history of the concept of sentience, and its use in policy and animal advocacy, though noting that we are inevitably faced with uncertainty regarding the subjective states of other animals. As we shall argue, however, their fears are overblown. Here, we add a suggestion we think might strengthen the discussion on feelings and welfare assessment. Drawing on evolutionary considerations about the functions of feelings will give us more reason for optimism and our goal here will be to outline an approach for how we could measure animal feelings.

Like Rowan et al. (2021), we agree that animal welfare consists in the feelings of animals – the positively and negatively valenced mental states that are consciously experienced (see Browning 2020 for a defence of this welfare concept). But while the authors think that research into animal feelings ‘brings with it a huge, almost insurmountable problem, which is that it is very difficult (and maybe impossible) to prove conclusively that any organism is sentient. Subjective feelings are just that— subjective—and are available only to the animal (or human) experiencing them’ (p.5), we contend that there is reason for more optimism.

We agree that measuring subjective feelings may be difficult, but not that this creates an insurmountable problem. After all, animal welfare science has spent a good part of the last two decades moving towards studying these experiences. The study of animal emotions is well-established (e.g. Désiré et al. 2002; Mendl and Paul 2004; Kremer et al. 2020), but the primary difficulty is still in distinguishing conscious, or felt, emotions, from the unconscious – a problem that has led some researchers to abandon the project entirely, in favour of other methods of assessing welfare (Dawkins 2021). However, we think there are ways to make progress on this question.

While it is true that feelings are subjective, we should expect them to have detectable causal effects that make a difference to lives of sentient creatures. As the authors rightly note, animal feelings have evolved to play a role in animals’ lives, i.e. by providing a fitness benefit (examples of plausible accounts can be found in Dawkins 1998; Fraser and  Duncan 1998; Veit 2022a, 2023). However, if Rowan et al. accept the common view that sentience provides animals with an evolutionary advantage, this would only have been possible if the presence of these feelings changes the animals’ phenotype in some way that is ‘visible’ to selection, for it is only actual causal impact of consciousness that could increase the survival and reproduction of such organisms such that we could think of consciousness as something that gradually evolved over evolutionary time. Such a view rules out the possibility of the feelings being epiphenomenal, i.e. a causally inefficacious by-product of other cognitive processes. This is important because natural selection does not invest in complex traits that have no adaptive function. If we think of consciousness as a mere by-product of cognitive processing, we would be unable to make sense of its obvious fit to the external (and internal) world in addition to its role in decision-making. If subjective experiences have causal effects, however, then – at least in theory – we will be able to study and measure them.

Adding the term ‘subjective’ to experiences may give off the impression that they are somehow distinct from the apparent ‘objective’ reality that the sciences investigate, but there is no such a thing as a magical boundary that divides the world of the material from the mental (see also Veit 2022b). We should not give in to these kinds of arguments that are sometimes used to undermine the objectives of animal ethics, legislation, and welfare science. Thus, the question shifts from if to how and should alleviate the scepticism that we will never be able to know even approximately what the experience of other animals is like. This isn’t to deny that animal consciousness is hard to study, but that there isn’t something mysterious about the phenomenon of subjective experience that makes it wholly unique from other phenomena that are hard to investigate.

Once we start building on the assumption that we can find ways of studying animal feelings by looking for the causal effects, we can broaden our empirical toolkit. Sceptics sometimes use slogans like one cannot infer a mental state from behaviour, but we have to distinguish between the claim that we can have absolute certainty about the experiences of others and the claim that no matter how much we learn about the brains, evolutionary history, and physiology of another animal that we cannot have any confidence about their mental states when confronted with a particular behaviour such as withdrawal from a needle or jumping behaviour when confronted with a new toy. No one is claiming that we can have certainty about the experiences of other animals. That is simply not how science works. Misattributions of feelings are possible without thereby implying that the entire field of research rests on mistaken assumptions. More evidence will increase our certainty about the possibility of feelings in different species as well as about what their actual experiences consist in.

Animal feelings will produce a range of detectable changes in neural processes, physiological functioning, and behaviour. Rowan et al. (2021) list a couple of approaches within the behavioural domain, including preference and motivation testing, and vocalisations. Beyond just the testing of how aversive (or pleasurable) an animal finds an experience, we may have means of assessing some of the qualitative features of these experiences – what it is like for the animal. We can develop tools for the identification of the presence and strength of different feelings in animals, based on their unique physical and behavioural signatures.

An example of this can be seen in the recent work on identifying markers of pain experience in cephalopod molluscs and decapod crustaceans (Birch et al. 2021; Crump et al. 2022). Beyond simply ascertaining the presence of sentience in these taxa, this work aimed to specifically identify a diverse set of physiological and behavioural markers that demonstrate the presence of pain experience, which could then be applied to identify this capacity in other taxa. A similar approach could be fruitful for other types of feelings. There has been a recent shift toward thinking about consciousness in terms of its dimensions rather than merely its presence or absence (Birch et al. 2020). The same is possible for an investigation of the valenced or ‘evaluative’ experiences of animals that matter for animal welfare. Rather than asking does an animal have feelings (i.e. is it sentient), we could instead be asking what feelings it has. As the research on animal sentience in understudied organisms (like crustaceans) has shown, a lack of evidence for sentience-related behaviours is often simply caused by the absence of relevant research. What is required is a deeply comparative approach across the animal tree of life that attempts to measure to quality and features of evaluative experience.

By developing such a mid-level approach that aims to generate a battery of tests and tools for measuring and assessing the range of animal feelings, we should be able to shape specific recommendations regarding policy, protections, and best-practice husbandry. As Rowan et al. note several times, it can be contested as to what the actual current impact has been of the recognition of sentience. While there are many potential ways recognition of sentience may have effects on treatment of animals (Browning and Veit 2022a, b), it is not yet clear to what degree this has been realised. While it is heartening to see the expansion of formal recognition of sentience in animal welfare and protection legislation around the world, it is unfortunately still unclear what this will mean for animals in practice. In particular, many of the animals used in agriculture have long been widely recognised as sentient, and yet still undergo a wide range of sufferings and deprivations. While a focus on recognition of sentience is important, it will only be effective if accompanied with real change in policy-making and our responses to animals. It is crucial not to let the recognition be merely symbolic and instead use this as the basis for advocating for better welfare protections for animals, with proper recognition of the empirical data on subjective wellbeing of these animals. Understanding the range and types of feelings an animal has the capacity to experience and, under what conditions, can thus help shape these more direct protections and ideally lead to improvements in animal welfare. But importantly, we want to emphasize that there is no reason to be pessimistic here. Science has best advanced by taking an optimistic approach towards complex challenges and we think the science of animal feelings can be similarly productive.
